# Global foot-and-mouth disease risk assessment based on multiple spatial analysis and ecological niche model

**DOI:** 10.1080/01652176.2025.2454482

**Published:** 2025-01-21

**Authors:** Qi An, Yiyang Lv, Yuepeng Li, Zhuo Sun, Xiang Gao, Hongbin Wang

**Affiliations:** a College of Veterinary Medicine, Northeast Agricultural University, Harbin, China; b Key Laboratory of the Provincial Education Department of Heilongjiang for Common Animal Disease Prevention and Treatment, College of Veterinary Medicine, Northeast Agricultural University, Harbin, China

**Keywords:** Foot-and-mouth disease, spatial epidemiology, risk assessment

## Abstract

Foot-and-Mouth Disease is a highly contagious transboundary animal disease. FMD has caused a significant economic impact globally due to direct losses and trade restrictions on animals and animal products. This study utilized multi-distance spatial cluster analysis, kernel density analysis, directional distribution analysis to investigate the spatial distribution patterns of historical FMD epidemics. A multi-algorithm ensemble model considering climatic, geographic, and social factors was developed to predict the suitability area for FMDV, and then risk maps of FMD for each species of livestock were generated in combination with the distribution of livestock. The results show that all serotypes of FMD exhibit significant clustering with a clear tendency toward a directional distribution. Serotypes A and O are widespread in Asia, Europe, Africa, and South America. Serotype Asia 1 is prevalent in Asia. Serotype SAT2 is prevalent in Africa and the Middle East, while Serotypes SAT1 and SAT3 are restricted to Africa. Ecological niche modeling reveals temperature, precipitation, wind speed, and vegetation are important factors influencing the occurrence of FMD. Except for buffaloes, the distribution of high-risk areas for FMD occurrence in other livestock species is quite widespread. The areas primarily include the southern region of North America, the northern, southern, and eastern regions of South America, the Mediterranean region, the eastern region of Europe, the central and southern regions of Africa, the central, eastern, and southern regions of Asia, and parts of Australia. These findings will provide valuable insights into the prevention and control of FMD.

## 1. Introduction

Foot-and-mouth disease (FMD) is a severe and highly contagious viral disease of cloven-hoofed animals caused by the foot-and-mouth disease virus (FMDV) (Grubman and Baxt 2004). FMDV is a single-stranded positive-sense RNA virus belonging to the *Aphthovirus* genus in the *Picornaviridae* family (Belsham 2005). Based on immunological characteristics, FMDV is classified into seven serotypes: A, Asia1, C, O, SAT1, SAT2, and SAT3 (Davies 2002). Each serotype is further divided into several subtypes (Song et al. 2014). There is no cross-immunity between different serotypes (Tekleghiorghis et al. 2016). Each serotype requires a specific vaccine to provide immunity for animals. The main prevalent serotypes of FMDV are A, Asia1, O, SAT1, SAT2, and SAT3, with C not reported since 2004 (Subramaniam et al. 2022). Serotypes A and O are the most widely distributed, and prevalent in Asia, Europe, Africa, and South America. Serotype Asia1 is primarily prevalent in Asia, while Serotypes SAT1 and SAT 3 are typically limited to Africa. Serotype SAT2 has spread from Southern Africa to Northern Africa and the Middle East (Jamal and Belsham 2013). FMD affects nearly all cloven-hoofed animals, including cattle, buffalo, goats, sheep, pigs, and more than 70 species of wild animals (Alexandersen and Mowat 2005). It is noteworthy that African buffalo (*Syncerus caffer*) can be persistently and asymptomatically infected with FMDV, which plays an important role in the maintenance and spread of FMD (Dion et al. 2011). Cattle carriers typically do not carry the virus for more than 6 months, while African buffalo may harbor FMDV for over 5 years (WOAH 2013). Infected livestock with FMDV may exhibit fever and develop vesicular lesions in the mouth, nose, feet, and teats (Alexandersen et al. 2003). FMD cannot be clinically distinguished from other vesicular diseases, such as vesicular stomatitis, vesicular exanthema, and swine vesicular disease (Grubman and Baxt 2004). From a clinical perspective, cattle are the most severely affected species, followed by water buffalo, while FMD in sheep and goats is typically subclinical (Subramaniam et al. 2022). FMDV may be present in all secretions and excretions of infected animals, including exhaled air (Brito et al. 2017; WOAH 2022). Under certain temperature conditions, FMDV can survive in the environment for several days to weeks (WOAH 2013). Susceptible animals may become infected with FMDV through direct contact or indirect contact. Direct contact is the most common transmission mode of FMD. Cattle and sheep can be infected at close range through inhalation of FMDV in aerosol form, while pigs are primarily infected through ingestion of contaminated animal products (Alexandersen et al. 2003). Indirect contact refers to the infection of susceptible animals by contact with FMDV carried by people, vehicles, and other objects (Valarcher et al. 2008). The long-distance spread of FMD is attributed to the movement of infected animals, the transport of FMDV contaminants by humans and vehicles, as well as the airborne transmission of FMDV through wind (Green et al. 2006).

As one of the most contagious animal diseases, FMD has an almost 100% morbidity rate, but a lower mortality rate (Radostits et al. 1994; Madin 2011). Large livestock infected with FMD experience growth suppression, reduced milk production, reduced fertility, and loss of traction (Knight-Jones and Rushton 2013; Munsey et al. 2019). Outbreaks of FMD can lead to significant economic losses due to direct losses and trade restrictions on animals and animal products, especially for developing countries dependent on the livestock industry (Baluka 2016). Countries with developed economies have been able to effectively control FMD, gradually establishing disease-free zones or even eradicating the disease. Countries with underdeveloped economies or unstable social order, where FMD occurs frequently, are still suffering severely. To reduce the impact of FMD worldwide, the Food and Agriculture Organization of the United Nations (FAO) and the World Organization for Animal Health (WOAH) jointly drafted a Global FMD Control Strategy based on the Global Framework for the Progressive Control of Transboundary Animal Diseases (GF-TADs) (Sun et al. 2022). The Global FMD Control Strategy consists of the Progressive Control Pathway for Foot and Mouth Disease (PCP-FMD) and the Performance of Veterinary Services (PVS) Pathway (Li et al. 2014). The PCP-FMD aims to assist countries affected by FMD outbreaks to improve risk management and gradually reduce the impact of FMD. The PVS Pathway aims to enhance the quality and capacity of national veterinary services to address the threat of animal diseases. FMD has shown a worldwide distribution in the past, with historical occurrences in Asia, Europe, Africa, the Americas, and Oceania (Song et al. 2014). Thanks to the efforts of FAO, WOAH, and countries in prevention and control measures, FMD has transitioned from a global distribution to a local regional distribution. The latest WOAH members’ official FMD status map shows that North America, Oceania, Europe, and most countries of South America have been recognized as being free of FMD (https://www.woah.org/en/disease/foot-and-mouth-disease/#ui-id-2, last accessed in October 2024). In certain regions of Asia and Africa, the prevalence of FMD remains high, and the scope of some dominant strains is gradually expanding (Sun et al. 2022). As a result, achieving the goal of FMD eradication in these regions continues to be a great challenge. Currently, the most widely used vaccine is based on inactivated FMDV, which has achieved tremendous success in controlling FMD globally (Cao et al. 2016). Although inactivated vaccines can help countries or regions achieve the FMD-free recognition of WOAH, inactivated vaccines suffer from the inability to distinguish between naturally infected animals and vaccinated animals (Parida 2009). The lack of DIVA (Differentiating Infected from Vaccinated Animals) vaccine calls into question the FMD status of animals in the region, and trade restrictions may remain in place (Smith et al. 2014). The FMD trade policies established by the WOAH encourage countries or regions to implement control measures in addition to vaccination, such as border controls, restrictions on the import of animals and animal products, and limitations on animal movement (Sumption et al. 2007; Barasa et al. 2008; Paton et al. 2010). These measures, while effective, significantly raise costs and still cannot eliminate the risk of virus circulation.

Spatial epidemiology is a powerful tool for exploring epidemiological patterns of disease and risk factors, it can analyze the impact of landscape structure and climatic conditions on the spread of infectious diseases (Dion et al. 2011). The ecological niche model based on the ecological niche theory is widely used in the fields of ecology such as species invasion and biodiversity conservation (Peterson and Vieglais 2001; Adhikari et al. 2019). In recent years, ENM has been used to predict animal infectious diseases such as lumpy skin disease (Li et al. 2023), equine infectious anemia (An et al. 2024), and peste des petits ruminants (Assefa et al. 2021), becoming a powerful tool for veterinary medicine to map the risk of infectious diseases. ENM explores the relationship between viruses and environmental variables through algorithms and calculates the environmental suitability of viruses in new time or space based on this relationship, thereby predicting the risk of disease occurrence (Escobar 2020). The objectives of this study include: (1) investigating the clustering phenomenon, degree of clustering, and distribution direction of historical FMD outbreaks; and (2) identifying the risk factors for FMD, and risk areas for FMD outbreaks in each species of livestock on a global scale. The findings of this study will provide valuable insights into the prevention and control of FMD, allowing for the optimization of resource allocation and utilization.

## 2. Materials and methods

### 2.1. FMD incidence data collection

The FMD incidence data was sourced from the Global Animal Disease Information System of the Food and Agriculture Organization of the United Nations (EMPRES-I+) (https://empres-i.apps.fao.org/). The data collection period spans from 1 January 2005, to 30 September 2023. The information collected included the time and location of the FMD epidemic, the species of animals infected, and the latitude and longitude coordinates. After cleaning and organizing the data, there were a total of 5,676 records remaining.

### 2.2. Spatial distribution characteristics of FMD

#### 2.2.1. Multi-distance spatial cluster analysis

Multi-distance spatial cluster analysis was performed on the FMD point data using Ripley’s K function to determine whether the FMDs exhibited statistically significant clustering or dispersion over a range of distances. The output of Ripley’s K contains the expected K value, the observed K value, and the confidence envelope. The confidence envelope is calculated by randomly placing 99 sets of points in the study area, with each set containing the same number of points as being analyzed. When the observed K value is greater than the expected K value and greater than the Higher Confidence Envelope, it means that FMD exhibits significant clustering at that distance. When the observed K value is less than the expected K value and less than the Lower Confidence Envelope, it means that FMD exhibits significant dispersion at that distance. Multi-distance spatial cluster analysis was completed in ArcGIS 10.2, the principles are detailed at https://desktop.arcgis.com/en/arcmap/latest/tools/spatial-statistics-toolbox/multi-distance-spatial-cluster-analysis.htm.

#### 2.2.2. Kernel density analysis

Kernel density analysis was used to explore the degree of FMD aggregation and to determine the high-incidence area of the disease. Kernel density analysis uses a kernel function to calculate a measure per unit area based on the FMD point data to fit each point to a smooth curved surface. Kernel density analysis outputs the results as a raster layer, with larger raster values for grids representing a greater density of FMD outbreaks in the vicinity. Kernel density analysis was completed in ArcGIS 10.2, the principles are detailed at https://desktop.arcgis.com/en/arcmap/latest/tools/spatial-analyst-toolbox/kernel-density.htm.

#### 2.2.3. Directional distribution analysis

The standard deviation ellipse method was employed to determine the spatial distribution characteristics of FMD. The standard deviation ellipse creates an ellipse surface based on the FMD point data, and the attributed values of the ellipse surface include the long and short axes, the mean center, and the rotation angle. Based on these attributed values, it can be determined whether the FMD has a specific directional distribution. The size of the ellipse was set to one standard deviation, containing about 68% of the FMD point data. Directional distribution analysis was completed in ArcGIS 10.2, the principles are detailed at https://desktop.arcgis.com/en/arcmap/latest/tools/spatial-statistics-toolbox/directional-distribution.htm.

### 2.3. Ecological niche modeling

#### 2.3.1. Filtering of FMD incidence data

To reduce the spatial autocorrelation between FMD incidence points, ENMTooLs software was performed for purification (Warren et al. 2010). This purification allows the FMD incidence data to match the resolution of the raster data used for modeling, ensuring that there is only one FMD occurrence point in each grid. Finally, the remaining 2558 records were used for subsequent modeling.

#### 2.3.2. Variable collection

Based on potential risk factors involved in the spread of FMD (Muroga et al. 2013; Jiang et al. 2020; Gao and Ma 2021), climate variables (temperature, precipitation, wind speed), topographic variables (elevation, land cover, NDVI), and social variables (population density, road density) were collected. The climatic variables, elevation, and wind speed were sourced from WorldClim 2.1 (https://www.worldclim.org/data/worldclim21.html). Bioclimatic variables are the average for the years 1970-2000. The wind speed was obtained by averaging the monthly wind speeds for the 12 months (January to December). The normalized difference vegetation index (NDVI) was downloaded from the National Tibetan Plateau Data Center (TPDC 2018). This dataset was produced by the NOAA Global Inventory Monitoring and Modeling System (GIMMS), with a temporal resolution of twice a month and a temporal coverage of 1981 to 2015. The maximum value composite method was used to obtain the average NDVI (Holben 1986; Julien and Sobrino 2019). Population density data was sourced from LandScan (https://landscan.ornl.gov/). The road density dataset was downloaded from the GRIP global roads database (https://www.globio.info/download-grip-dataset), which includes a combination of five types of roads. The land cover data comes from EarthEnv’s Global 1-km Consensus Land Cover (https://www.earthenv.org/landcover), which provides consensus information on the prevalence of 12 land cover classes. All raster have a resolution of 5 arc minutes and a coordinate system of WGS 1984. The usdm package of the R software was used to screen for climatic and land cover variables, with criteria of a VIF value less than 5 and the absolute value of Pearson correlation coefficients less than 0.7. In addition, some variables were removed based on ecological significance and their contribution to the model. The final variables used for modeling are shown in Table 1.

**Table 1.** Variables used in the model.

Code	Variable name	Source
bio8	Mean Temperature of Wettest Quarter	WorldClim version 2.1
bio10	Mean Temperature of Warmest Quarter	WorldClim version 2.1
bio15	Precipitation Seasonality	WorldClim version 2.1
class1	Evergreen/Deciduous Needleleaf Trees	EarthEnv
class7	Cultivated and Managed Vegetation	EarthEnv
people	Population Density	LandScan
wind	Wind Speed	WorldClim version 2.1
road	Road Density	GRIP global roads database
ndvi	Normalized Difference Vegetation Index	National Tibetan Plateau Data Center

#### 2.3.3. Modeling procedure

In this study, the ecological niche modeling of FMD was conducted using the biomod2 package in R (Thuiller et al. 2016). Biomod2 contains a variety of modeling techniques and allows the combination of different models into an ensemble model. The Surface Range Envelope (SRE) method was used to generate five sets of pseudo-absence points. Each set of pseudo-absence points was three times the number of FMD incidence points. Ten algorithms were selected to build individual models for FMD, including Artificial Neural Network (ANN), Classification Tree Analysis (CTA), Flexible Discriminant Analysis (FDA), Generalized Additive Model (GAM), Generalized Boosting Model (GBM), Generalized Linear Model (GLM), Multiple Adaptive Regression Splines (MARS), Maximum Entropy (MAXENT), Random Forest (RF), and eXtreme Gradient Boosting Training (XGBOOST). For each presence-pseudo-absence dataset, each algorithm was repeated ten times to build a total of 500 FMD individual models (5*10*10). For each model, 80% of the data is used for training the model, while the remaining 20% is used for testing the model. The parameter optimization strategy for individual models was set to ‘bigboss’. The model performance was evaluated using, AUC, KAPPA, and TSS. Individual models with a TSS greater than 0.8 entered the final ensemble process. The ensemble rule is the weighted average, where the higher the TSS value of an individual model, the greater its proportion in the ensemble process. The final ensemble model was used to predict the suitability areas for FMDV.

### 2.4. Quantifying areas of exposure risk for livestock

Susceptible livestock for FMD include cattle, buffaloes, goats, sheep, and pigs. Gridded data for these five species of livestock were downloaded from the FAO Livestock System (https://www.fao.org/livestock-systems/en/). Gridded data for each species of livestock was divided into four categories: 0, 1, 2, and 3, corresponding to densities of (0, 1) animal/km^2^, [1, 10) animals/km^2^, [10, 100) animals/km^2^, and ≥ 100 animals/km^2^ respectively. The FMDV suitability map generated by the ensemble model was classified at equal intervals into 4 categories (0, 1, 2, 3). The classification density map for each species of livestock and the classification FMDV suitability map were multiplied together using a raster calculator to produce a risk map of FMD occurrence in each species of livestock. The risk of FMD occurrence in each species of livestock was classified into five levels, namely very low, low, moderate, high, and very high.

## 3. Results

### 3.1. Spatial characteristics of FMD

**Figure 1.**
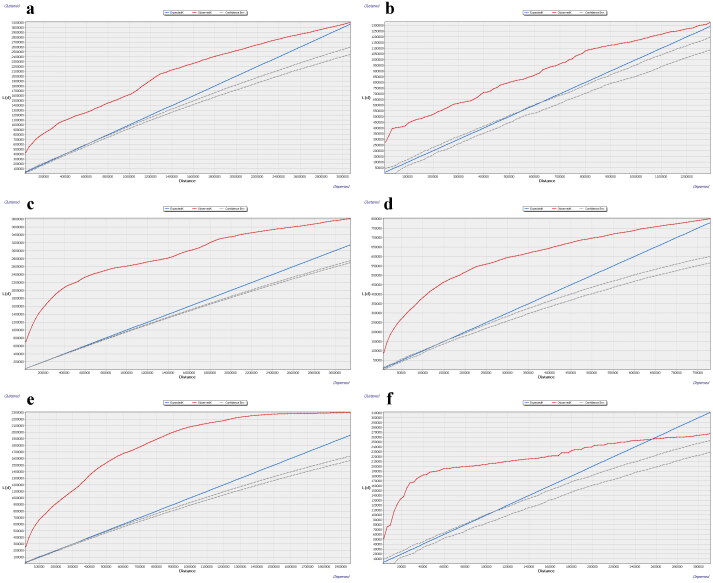
Results of multi-distance spatial cluster analysis of six serotypes of FMD. (a) A, (b) Asia1, (c) O, (d) SAT1, (e) SAT2, (f) SAT3.

**Figure 2.**
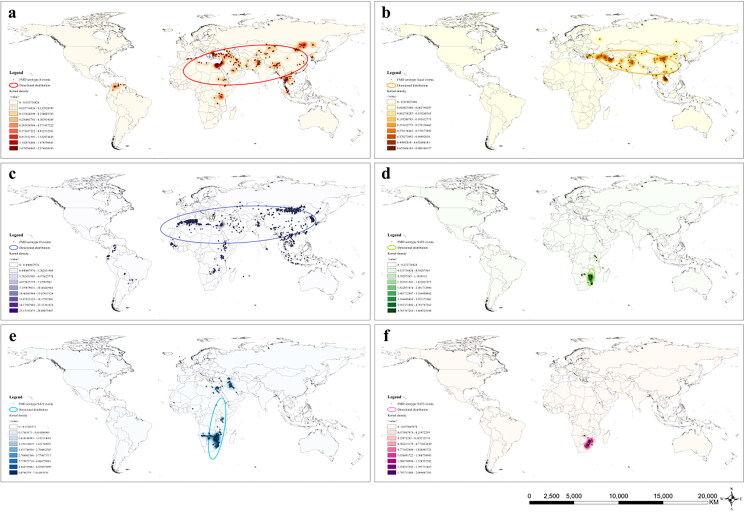
Results of the directional distribution analysis and kernel density analysis of the six serotypes of FMD. (a) A, (b) Asia1, (c) O, (d) SAT1, (e) SAT2, (f) SAT3.

From the results of multi-distance spatial cluster analysis, serotype A, Asia1, O, SAT1, and SAT2 all exhibit significant clustering within the set distance range (Figure 1). The serotype SAT3 is significantly clustered below approximately 256,000 meters. When the distance exceeds 256,000 meters, the observed K value for serotype SAT3 is lower than the expected K value curve but higher than the Lower Confidence Envelope. Therefore, the result is not statistically significant. The geographical distribution of the six serotypes of FMD from 2005 to September 2023 is shown in Table S1. In decreasing order of the number of countries affected, these are serotypes O, A, SAT2, Asia1, SAT1, and SAT3. The results of kernel density analysis and directional distribution analysis clearly show the hotspot areas and distribution directions of the six serotypes. Serotype A is distributed in Asia, Europe, Africa, and South America, with a northeast-southwest distribution direction (Figure 2). Hotspot areas (with a high degree of aggregation) include the coast of the Marmara Sea, the southeast coast of the Mediterranean, southern Thailand, and the region bordered by the eastern of Mongolia, China, and Russia. Serotype Asia1 is primarily distributed in Asia in a northwest-southeast distribution direction, with hotspot areas in eastern Turkey, northwestern Iran, central China, southern Laos, and central Vietnam. Serotype O is the most widely distributed, spanning Asia, Europe, Africa, and South America, with an overall northeast-southwest distribution direction. The south-western coastal region and south-eastern coastal region of the Mediterranean Sea, the Korean Peninsula, and south-western Japan are hotspot areas for Serotype O. Serotype SAT1 is similar to Serotype SAT3 in that it is confined to southern Africa and has a northeast-southwest distribution direction. The difference is that the hotspot area for the Serotype SAT1 is southern Zimbabwe and the hotspot area for the Serotype SAT3 is north-eastern South Africa. In addition to southern Africa, Serotype SAT2 is also distributed in the Middle East region. The distribution direction of Serotype SAT2 is northeast-southwest, and the hotspot areas are in Zimbabwe and northeastern South Africa.

### 3.2. Evaluation results of FMD individual models and ensemble model

Table 2 shows the average evaluation results of fifty runs of each algorithm. Combining the three types of evaluation metrics, Random Forest performed the best (AUC, KAPPA, and TSS of 0.98, 0.82, and 0.83, respectively) and Generalized Additive Model performed the worst (AUC, KAPPA, and TSS of 0.85, 0.48, and 0.56, respectively). High-precision individual models (TSS > 0.8) were chosen to build the ensemble model. The ensemble model AUC, KAPPA, and TSS were 0.99, 0.83, and 0.89, respectively, indicating excellent performance of the ensemble model.

**Table 2.** Evaluation results of FMD individual models.

Evaluation	ANN	CTA	FDA	GAM	GBM	GLM	MARS	MAXENT	RF	XGBOOST
AUC	0.93	0.95	0.94	0.85	0.97	0.95	0.95	0.96	0.98	0.93
KAPPA	0.68	0.79	0.70	0.48	0.80	0.73	0.74	0.74	0.82	0.71
TSS	0.74	0.81	0.72	0.56	0.81	0.74	0.77	0.81	0.83	0.73

### 3.3. Important variables of the ensemble model and their response curves

**Figure 3.**
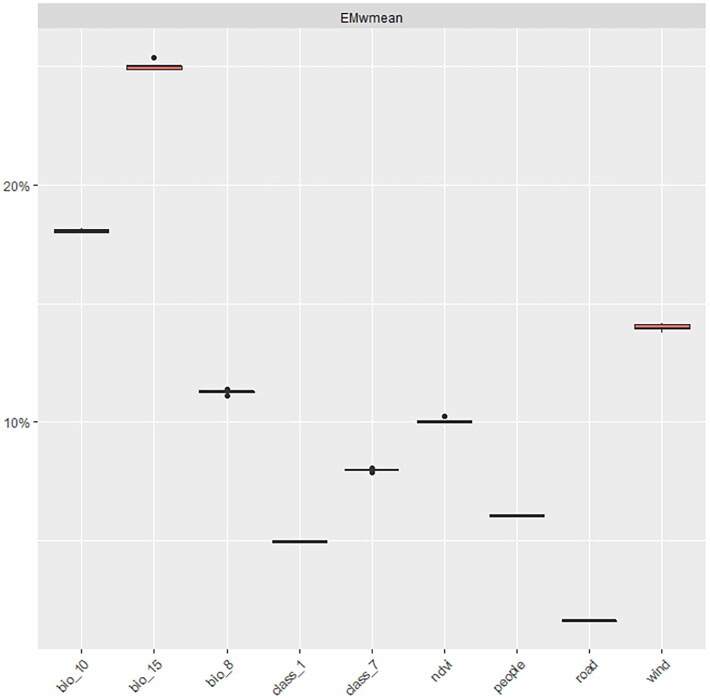
The relative importance of variables in the FMD ensemble model. The results were calculated through five permutations.

**Figure 4.**
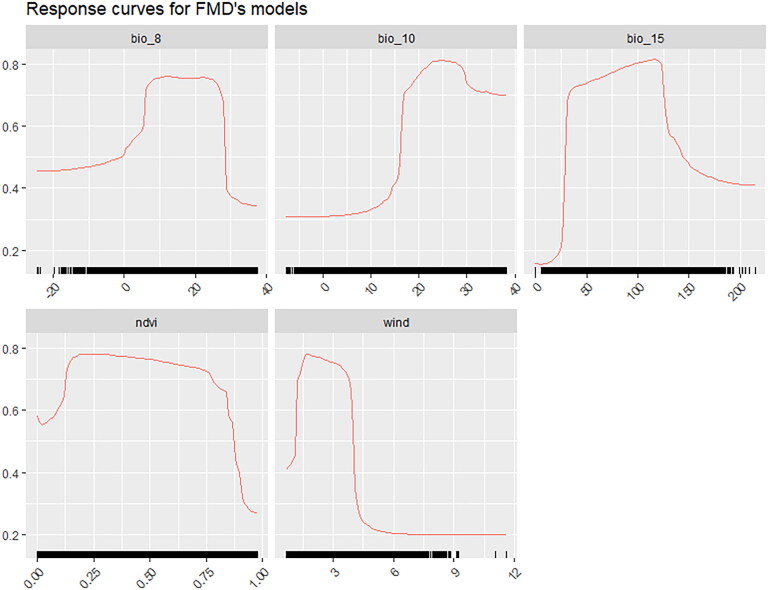
Response curves for important variables in the FMD ensemble model. Bio8 represents mean temperature of wettest quarter, bio10 represents mean temperature of warmest quarter, bio15 represents precipitation seasonality, wind represents wind speed, NDVI represents normalized difference vegetation index.

Figure 3 illustrates the relative contribution of the variables in the FMD ensemble model, calculated as the average of 5 permutations. Variables with relative contributions greater than or equal to 10% were selected as important variables influencing the FMD occurrence, including mean temperature of warmest quarter (bio10), precipitation seasonality (bio15), wind speed (wind), mean temperature of wettest quarter (bio8) and normalized difference vegetation index (NDVI). From Figure 4, it is evident that FMDV is suited to survive in warm environments with some vegetation and less seasonal variation in precipitation.

### 3.4. Risk map of FMD occurrence in livestock

**Figure 5.**
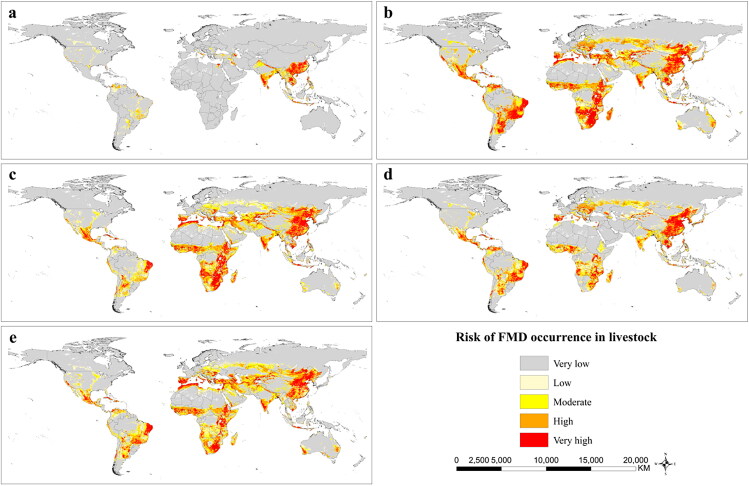
Risk map of FMD occurrence in five species of livestock. The grey color depicts very low risk, the light yellow color depicts low risk, the yellow color depicts moderate risk, the orange color depicts high risk, and the red color depicts very high risk. (a) buffaloes, (b) cattle, (c) goats, (d) pigs, (e) sheep.

Figure 5 reveals the global risk areas where five species of livestock are exposed to FMD. The high-risk areas of FMD occurrence in buffaloes primarily include scattered regions of the Americas, southern Italy, parts of the Middle East, parts of South Asia, parts of Southeast Asia, and southern China. The high-risk areas of FMD occurrence in cattle, goats, pigs, and sheep are similar and primarily include the southern region of North America, the northern, southern, and eastern regions of South America, the Mediterranean region, the eastern region of Europe, the central and southern regions of Africa, the central, eastern, and southern regions of Asia, and parts of Australia.

## 4. Discussion

FMD is a legally notifiable infectious disease and also a transboundary animal disease. FMD has caused a significant economic impact worldwide over the past decades due to direct losses and trade restrictions on animals and animal products. Although many countries have achieved FMD-free status without vaccination, they still face the threat of invasion. In response to this situation, this study utilized multiple spatial analysis techniques and ecological niche modeling to investigate the spatial distribution patterns of the historical FMD epidemic and predict the risk areas of FMD occurrence in five species of livestock, to provide a reference for decision-makers. The results of the multi-distance spatial cluster analysis indicate that almost all serotypes of FMD show significant clustering patterns within a specified distance range, which could be attributed to multiple factors. Specific climate and environmental conditions, the activity patterns and aggregation behavior of cloven-hoofed animals, the effectiveness and coverage of vaccines, and human activities such as trade and transportation may all contribute to the clustering of FMD. The results of directional distribution analysis provide insights into the distribution of six serotypes and the overall direction of their spread and diffusion. The flatness of the standard deviation ellipse (the ratio of the difference between the long and short half-axes of the ellipse to the long half-axis) was large for all six serotypes, showing a clear tendency toward a directional distribution (Table S2). Serotypes A, O, SAT1, SAT2, and SAT3 all exhibit a northeast-southwest distribution direction, with different rotation angles. Serotype Asia1 exhibits a northwest-southeast distribution direction. Based on genetic relationships and antigenic analyses, the WOAH/FAO Reference Laboratory classified global FMDV into seven virus pools (Song et al. 2015). The division of the viral pool indirectly reflects animal movement, trade patterns, and the distribution of wild hosts (Jamal and Belsham 2013). In recent years, FMDV has shown a tendency to spread to new regions, with the epidemic range of some strains gradually expanding. Some of the dominant strains have demonstrated clear trans-pool transmission (Jamal and Belsham 2018; Pezzoni et al. 2019). Long-distance transmission of FMD plays an important role in this epidemic pattern, and possible modes of transmission include the movement of infected animals, transport of FMD contaminants, and airborne transmission (Green et al. 2006).

To quantify the risk areas of FMD occurrence and to explore the risk factors that may influence FMD occurrence, ecological niche models were developed using FMD incidence data, climatic variables, topographic variables, and social variables. Most previous studies have only considered a single modeling technique to predict the distribution of a disease (Jiang et al. 2020; Gao and Ma 2021). Research has shown that ensemble models exhibit higher accuracy and better performance compared to individual models (Čengić et al. 2020). Ensemble models use specific rules to combine multiple individual models into a prediction model, which can reduce model error (Leta et al. 2019). Ensemble models can produce accurate predictions even with incomplete data (Deka et al. 2022). The model evaluation results show that the evaluation metrics of the ensemble model are significantly higher than those of individual models, revealing its excellent performance. The Random Forest model performed the best among individual models, likely due to the unique Bagging approach, which enhances classification accuracy and generalization ability (Parmar et al. 2019).

The results of the variable importance in the FMD ensemble model show that the mean temperature of warmest quarter, precipitation seasonality, wind speed, mean temperature of wettest quarter and normalized difference vegetation index are the important variables affecting the occurrence of FMD. The response curves of the mean temperature of warmest quarter and mean temperature of wettest quarter reveal that FMDV is more suited to warmer environments. FMDV is progressively inactivated above 50 °C (WOAH 2013). Excessive temperatures may be detrimental to the survival of FMDV in the environment (Subramaniam et al. 2022). It is important to note that mean temperature of warmest quarter and mean temperature of wettest quarter are the average of temperatures over a quarter, and the specific temperatures suitable for FMDV are subject to further research. From the response curves of precipitation seasonality, it can be observed that FMDV prefers to lower precipitation seasonality. Precipitation makes the air more humid, which may provide suitable conditions for the survival and spread of FMDV in the environment (Gloster et al. 2003; Kitching et al. 2005; Hossain et al. 2023). Hagerman and colleagues have revealed that moderate weather conditions, without extreme temperatures or precipitation, are conducive to the airborne transmission of FMDV (Hagerman et al. 2018). The response curve of wind speed shows that FMDV has higher suitability under lower wind speed conditions. A possible explanation is that low-speed breezes are favorable for the airborne transmission of FMDV (Donaldson and Alexandersen 2002; Alexandersen and Mowat 2005), while strong winds with higher speeds can reduce the concentration of viruses in aerosols (Sutmoller et al. 2003). In addition, the response curves show that the FMDV maintains high suitability over a wide range of NDVI variations. The susceptible host may play an important role in this. NDVI represents the degree of vegetation coverage, and a suitable NDVI provides sufficient food for wild animals and grazing livestock.

In an era of globalization and increased global travel, trade, and transport of goods, FMD remains a permanent threat to all countries. Although most countries in North America, Oceania, Europe, and most countries of South America have been recognized as FMD-free by WOAH, parts of these regions still have suitable conditions for FMD occurrence and need to be alert to the risk of FMD introduction. In recent years, FMD has spread widely in Asia and Africa, with severe outbreaks in some countries and regions, causing significant economic losses (Souley Kouato et al. 2018b; Giasuddin et al. 2020; Aslam and Alkheraije 2023; Zewdie et al. 2023). The official global FMD control strategy and regional control strategies (such as South-East Asia and China Foot and Mouth Disease Campaign) should continue to be pursued in these areas to progressively improve the epidemic status of FMD.

This research has certain limitations. The FMD epidemic data used in this study all come from official reports. If there are any underreported epidemics, research findings may deviate from the true epidemiology of FMD. In addition, animal movement was not considered in this study due to the lack of relevant data. Animal movement is commonly reported as a risk factor in the literature and likely plays a significant role in the transmission and spread of FMD (Mikkelsen et al. 2003; Souley Kouato et al. 2018a). Another point is that wild animals were not considered in this study. As a multi-species disease, FMD can infect almost all cloven-hoofed animals, including over 70 species of wild animals (Alexandersen and Mowat 2005). Wild animals are the primary vector for the maintenance and spread of FMD in nature. Contact between African buffalo and domestic cattle at water points and grazing areas often leads to outbreaks of FMD in domestic cattle (Dion et al. 2011). However, there is a lack of available information on the global distribution of wild hosts of FMD.

## 5. Conclusion

All serotypes of FMD showed significant clustering with a clear tendency toward a directional distribution. Ecological niche modeling reveals temperature, precipitation, wind speed, and vegetation are important factors influencing the occurrence of FMD. Except for buffaloes, the distribution of high-risk areas for FMD occurrence in other livestock species is quite widespread. The areas primarily include the southern region of North America, the northern, southern, and eastern regions of South America, the Mediterranean region, the eastern region of Europe, the central and southern regions of Africa, the central, eastern, and southern regions of Asia, and parts of Australia. This study has strategic significance for the prevention and control of FMD.

## Supplementary Material

Supplementary Materials.docx

## Data Availability

The datasets generated and/or analyzed during the current study are available from the corresponding author upon reasonable request.
