# Supplementary material for: Global foot-and-mouth disease risk assessment based on multiple spatial analysis and ecological niche model
Source: Vet Q. 2025 Jan 21;45(1):1–11. doi: 10.1080/01652176.2025.2454482 (PMC11755741; doi:10.1080/01652176.2025.2454482)
Supplement: Supplementary Materials.docx [file TVEQ_A_2454482_SM7880.docx]

Table S1. Geographical distribution of six serotypes of FMD, 2005 to September 2023.

| Serotype | Country | Number of outbreak |
| --- | --- | --- |
| A | Afghanistan | 6 |
|  | Algeria | 5 |
|  | Armenia | 2 |
|  | Bahrain | 2 |
|  | Bhutan | 1 |
|  | China | 42 |
|  | Colombia | 8 |
|  | Democratic Republic of the Congo | 3 |
|  | Egypt | 60 |
|  | Eritrea | 6 |
|  | Ethiopia | 3 |
|  | Gaza Strip | 3 |
|  | Iran | 27 |
|  | Iraq | 2 |
|  | Israel | 20 |
|  | Jordan | 2 |
|  | Kazakhstan | 11 |
|  | Kenya | 2 |
|  | Kyrgyzstan | 2 |
|  | Lao People's Democratic Republic | 1 |
|  | Libya | 13 |
|  | Malaysia | 11 |
|  | Mongolia | 25 |
|  | Myanmar | 4 |
|  | Nepal | 2 |
|  | Pakistan | 19 |
| A | Republic of Korea | 10 |
|  | Russian Federation | 48 |
|  | Saudi Arabia | 10 |
|  | Sudan | 5 |
|  | Thailand | 38 |
|  | Tunisia | 2 |
|  | Turkey | 56 |
|  | Uganda | 1 |
|  | United Republic of Tanzania | 17 |
|  | Venezuela | 13 |
|  | Viet Nam | 11 |
|  | West Bank | 1 |
|  | Zambia | 2 |
| Asia 1 | Afghanistan | 5 |
|  | Bahrain | 1 |
|  | China | 47 |
| Asia 1 | Dem People's Rep of Korea | 2 |
|  | Iran | 27 |
|  | Lao People's Democratic Republic | 1 |
|  | Myanmar | 4 |
|  | Nepal | 2 |
|  | Pakistan | 13 |
|  | Russian Federation | 4 |
|  | Tajikistan | 2 |
|  | Turkey | 20 |
|  | Viet Nam | 20 |
| O | Afghanistan | 3 |
|  | Algeria | 986 |
|  | Argentina | 5 |
|  | Bahrain | 1 |
|  | Bhutan | 5 |
|  | Bolivia | 7 |
|  | Brazil | 47 |
|  | Bulgaria | 23 |
|  | Cambodia | 6 |
|  | China | 127 |
|  | Colombia | 30 |
|  | Comoros | 1 |
| O | Cyprus | 3 |
|  | Dem People's Rep of Korea | 116 |
|  | Democratic Republic of the Congo | 1 |
|  | Ecuador | 15 |
|  | Egypt | 20 |
|  | Eritrea | 5 |
|  | Ethiopia | 33 |
|  | Gambia | 3 |
|  | Gaza Strip | 7 |
|  | Georgia | 1 |
|  | Guinea | 7 |
|  | Guinea-Bissau | 56 |
|  | Indonesia | 56 |
|  | Iran | 16 |
|  | Iraq | 4 |
|  | Israel | 281 |
|  | Japan | 302 |
|  | Jordan | 21 |
|  | Kazakhstan | 36 |
|  | Kenya | 23 |
|  | Kuwait | 16 |
|  | Kyrgyzstan | 1 |
| O | Lao People's Democratic Republic | 14 |
|  | Libya | 116 |
|  | Malawi | 2 |
|  | Malaysia | 1 |
|  | Mauritania | 2 |
|  | Mauritius | 13 |
|  | Mongolia | 387 |
|  | Morocco | 87 |
|  | Mozambique | 2 |
|  | Myanmar | 22 |
|  | Namibia | 3 |
|  | Nepal | 5 |
|  | Pakistan | 40 |
|  | Paraguay | 4 |
|  | Republic of Korea | 260 |
|  | Russian Federation | 70 |
|  | Saudi Arabia | 5 |
|  | South Sudan | 1 |
|  | Sri Lanka | 7 |
|  | Sudan | 4 |
|  | Syrian Arab Republic | 4 |
|  | Thailand | 21 |
| O | Tunisia | 321 |
|  | Turkey | 9 |
|  | U.K. of Great Britain and Northern Ireland | 18 |
|  | United Arab Emirates | 3 |
|  | United Republic of Tanzania | 2 |
|  | Venezuela | 5 |
|  | Viet Nam | 116 |
|  | West Bank | 49 |
|  | Zambia | 22 |
| SAT1 | Botswana | 7 |
|  | Comoros | 1 |
|  | Kenya | 9 |
|  | Namibia | 8 |
|  | South Africa | 74 |
|  | United Republic of Tanzania | 9 |
|  | Zimbabwe | 141 |
| SAT2 | Angola | 5 |
|  | Bahrain | 3 |
|  | Botswana | 93 |
|  | Egypt | 66 |
|  | Gaza Strip | 2 |
| SAT2 | Iraq | 74 |
|  | Jordan | 1 |
|  | Kenya | 11 |
|  | Libya | 2 |
|  | Malawi | 10 |
|  | Mauritania | 1 |
|  | Mozambique | 15 |
|  | Namibia | 36 |
|  | Nigeria | 2 |
|  | South Africa | 205 |
|  | Sudan | 1 |
|  | Turkey | 10 |
|  | United Republic of Tanzania | 6 |
|  | Zambia | 6 |
|  | Zimbabwe | 246 |
| SAT3 | Namibia | 5 |
|  | South Africa | 103 |
|  | Zimbabwe | 1 |

Table S2. The attributed values for standard deviation ellipse of global FMD epidemics, 2005 to September 2023.

| Serotype | Center coordinates | | XStdDist | YStdDist | Rotation |
| --- | --- | --- | --- | --- | --- |
| A | | (59.673898, 29.905552) | 19.098212 | 63.037822 | 83.799647 |
| Asia1 | | (78.857604, 32.649157) | 40.921357 | 11.085886 | 97.749658 |
| O | | (52.316332, 32.023223) | 18.317583 | 78.552023 | 86.616046 |
| SAT1 | | (31.156048, -20.808027) | 3.308958 | 8.621071 | 9.129311 |
| SAT2 | | (31.0452, -10.466305) | 7.363508 | 31.324597 | 9.059237 |
| SAT3 | | (28.796303, -25.06715) | 2.405049 | 4.252174 | 20.617995 |
